# Talimogene Laherparepvec combined with anti-PD-1 based immunotherapy for unresectable stage III-IV melanoma: a case series

**DOI:** 10.1186/s40425-018-0337-7

**Published:** 2018-05-16

**Authors:** Lillian Sun, Pauline Funchain, Jung Min Song, Patricia Rayman, Charles Tannenbaum, Jennifer Ko, Michael Mcnamara, C. Marcela Diaz-Montero, Brian Gastman

**Affiliations:** 1 Cleveland Clinic Lerner College of Medicine, Case Western Reserve University, Cleveland, 44106 OH USA; 2 Taussig Cancer Institute, Cleveland Clinic, Cleveland, 44195 OH USA; 3 Department of Immunology, Cleveland Clinic Lerner Research Institute, Cleveland, 44195 OH USA; 4 Departments of Pathology and Dermatology, Cleveland Clinic Foundation, Cleveland, 44195 OH USA; 5 Department of Plastic Surgery, Cleveland Clinic, Cleveland, 44195 OH USA; 6 NE4-303, Cleveland Clinic Lerner Research Institute, 9500 Euclid Ave, Cleveland, 44195 OH USA

## Abstract

**Background:**

Talimogene Laherparepvec (T-VEC) is an oncolytic virus approved as an intratumoral therapy for treating unresectable stage IIIB-IV metastatic melanoma. The mechanisms of action for T-VEC and checkpoint inhibitor are highly complementary. Recent studies have shown that combining checkpoint inhibitor therapy with T-VEC injection can lead to improved response rates for stage IIIB-IV melanoma patients.

**Methods:**

We reviewed 10 consecutive cases of stage IIIC to stage IVM1b melanoma patients that received T-VEC plus checkpoint inhibitor(s) therapy (pembrolizumab, ipilimumab/nivolumab, or nivolumab) treated between June 2016 and August 2017 at the Cleveland Clinic with a median follow-up of 7 months (range: 4 to 13 months). Responses of injected (on-target) and uninjected (off-target) lesions were evaluated according to RECIST 2.0.

**Results:**

The overall response rate for on-target lesions was 90%, with 6 patients experiencing a complete response in injected lesions. Two patients had off-target lesions, which were completely resolved after treatment. Blood samples were tested for 3 complete responders and 2 partial responders. CD4:CD8 ratio and frequencies of circulating PD1^+^ CD4 and CD8 T cells were elevated in complete responders but not partial responders. One patient died due to causes unrelated to melanoma and one patient died of progression of the disease.

**Conclusion:**

Our data suggest that combining checkpoint inhibitor(s) with T-VEC injection may provide a synergistic efficacy for patients with unresectable melanoma. We observed a better overall response rate and complete response rate compared to published studies on similar therapeutic regimens.

## Background

Talimogene laherparepvec (T-VEC) is an attenuated replication-competent herpes simplex virus type 1 (HSV-1) that selectively replicates in and lyses tumor cells [1]. T-VEC is approved as a treatment for patients with stage IIIB-IV metastatic melanoma as an intralesional injection. Data analysis from the phase III OPTiM trial of T-VEC monotherapy in patients with unresectable stages IIIB-IV melanoma showed a modest efficacy in treating advanced stage melanoma [2–6].

In addition to its oncolytic effect, T-VEC is also designed to elicit anti-tumor response by releasing tumor-associated antigens and providing cytokine stimuli via the local production of human granulocyte macrophage colony-stimulating factor (GM-CSF) encoded by the virus [6]. The combination of tumor destruction, release of tumor antigens with local GM-CSF expression can enhance tumor antigen presentation to T cells and subsequently promote the induction of anti-tumor immune responses [7]. This proposed mechanism of action for T-VEC is complementary to checkpoint inhibitor-based tumor immunotherapies such as the blockade of PD-1/PD-L1 or CTLA4, which helps the effector T cells to overcome negative regulation during priming and the effector stage. Hence, the combinatorial use of T-VEC and checkpoint inhibitors may achieve synergistic efficacy especially in the control of systemic disease [8]. In support of this idea, Phase Ib trials evaluating the combination of T-VEC with a checkpoint inhibitor therapy (anti-CTLA4 or anti-PD1) have reported higher overall response rates (ORR) and complete response rates (CRR) compared to historical data of T-VEC or checkpoint inhibitor monotherapy [2, 9].

We reviewed a case series of stage III-IVM1b melanoma patients treated with T-VEC injections in addition to pembrolizumab, nivolumab, or nivolumab plus ipilimumab. We observed an overall response rate (ORR) of 90%, with 60% of the patients achieving complete response (CR) in this cohort.

## Methods

### Patients characteristics

**Fig. 1:**
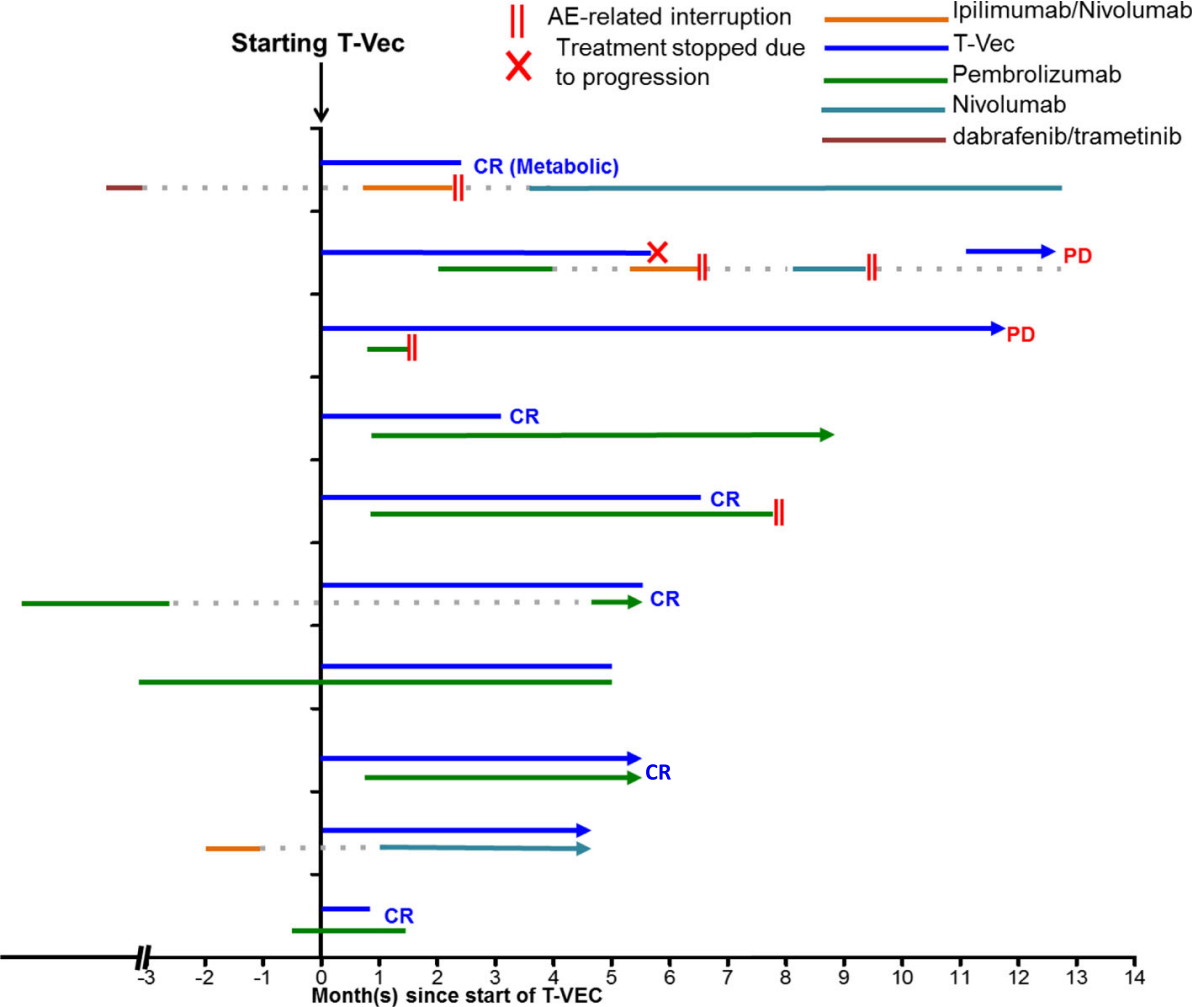
Timing of treatments. The time of T-VEC injection is set at 0. Types of treatment is indicated with different colors and durations of treatment is represented by the length of the line for each patient. II denotes suspension of treatment as a result of treatment-related adverse events. X denotes cessation of treatment due to progressive disease. AE, adverse events

A total of 10 consecutive patients with unresectable stage IIIC to stage IVM1b melanoma treated with an off-label use of T-VEC and checkpoint inhibitor therapy between June 2016 and August 2017 at Cleveland Clinic were analyzed. Median age at the time of the treatment was 73.5 (range 51–82). At the start of the treatment, all patients had an ECOG (Eastern Cooperative Oncology Group) score of less than 2 (Table 1). All patients included in the cohort had recurrent or residual disease after initial surgical treatment. Eight patients had unresectable stage III disease while 2 patients had pulmonary nodules that were consistent with metastases in the lung (stage IV, Table 1). Six of the patients included in this report were treatment naïve (Fig. 1). Three patients had prior exposure to checkpoint inhibitor therapy and 1 patient had received targeted therapy (Table 1, Fig. 1). Cleveland Clinic is a participating center for the MASTERKEY-265 study. Patients included in this case review either started treatment before the trial or failed to meet the inclusion criteria. T-VEC injection was performed in addition to the standard of care immunotherapy after extensive consultation with the patients and family. Treatment plans were also discussed at the melanoma tumor board.

**Table 1:** Patient Characteristics and Response Rates

Consecutive Patients (*n* = 10)	
Sex	Male = 7, Female = 3
Median Age (range)	70 (52–82)
Follow-up Time	4.5–13.6 Months
ECOG Performance Score	0 = 5, 1 = 4, 2 = 1
Patients with Distant Metastases	2
Unresectale Stage III	8
Stage IV	2
Prior exposure to immunotherapy	(1 adjuvant therapy, 2 systemic therapies)
Prior exposure to targeted therapy	1
Overall Response Rate (On-target lesions)	9/10
Overall Response Rate(Off-target lesions)	2/2

### Flow cytometric analysis of peripheral blood T cells

After obtaining consent for an institutional review board approved protocol, peripheral blood was collected in EDTA-coated tubes and subjected to density-gradient centrifugation using Ficoll-Paque to isolate peripheral blood isolate mononuclear cells (PBMCs). Freshly isolated PBMCs were stained and analyzed on a BD LSRFortessa™. Flow cytometric analysis for CD4, CD8 and PD1 was gated on viable cell population using on forward and side scatter.

### Evaluation of response

Responses of lesions that received direct T-VEC injection were considered “on-target”; responses of distant metastases that were not injected were designated “off-target”. Changes in tumor burden were assessed by extracting tumor information in the medical records, including both the clinical notes and imaging reports. Subcutaneous lesions and lung metastases were assessed based on PET and CT scan. The tumor response is expressed as change in size from baseline according to the Response Evaluation Criteria in Solid Tumors (RECIST) version 2.0 by investigator review.

## Results

### Treatment and adverse events

**Fig. 2:**
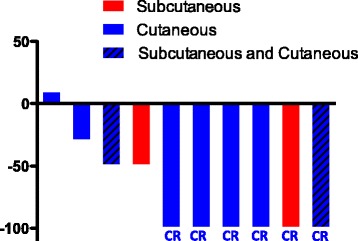
Best Response of on-target lesions. Changes in the injectable lesions for each patient are shown as waterfall plot. CR, complete response. Cutaneous lesions are shown in blue and subcutaneous lesions are shown in red. CR, complete response

The treatment protocol adopted for patients in our cohort was slightly different from that of the MASTERKEY-265 protocol in which T-VEC was administered on day 1 of weeks 4 and 6 and every 2 weeks thereafter. In our cohort, all patients received a lower dose of T-VEC for the first injection as recommended by manufacturer. After the initial dose, T-VEC was injected every 3 weeks until complete resolution of the injectable lesions or until the patient declined the treatment (Fig. 1). Six patients received injection in only cutaneous lesions (Fig. 2). Two patients had injection in subcutaneous lesions (Fig. 2). The other 2 patients received injection in both cutaneous and subcutaneous lesions (Fig. 2). In addition to T-VEC injection, all 10 patients were also treated with checkpoint inhibitor therapy (Fig. 1). Unlike the treatment protocol of MASTERKEY-265, in which patients received the first dose of pembrolizumab 6 weeks after the start of T-VEC, timing of checkpoint inhibitor treatment varied for patients in our cohort (Fig. 1). A total of 4 patients initiated checkpoint inhibitor therapy prior to the start of T-VEC injection. Two patients had been previously treated with ipilimumab plus nivolumab or pembrolizumab (Fig. 1); for both patients the treatments had been suspended due to adverse events and then resumed after T-VEC started (Fig. 1). Two patients had been treated with pembrolizumab as systemic therapy for at least a month when T-VEC injection was initiated (Fig. 1). In summary, eight patients received pembrolizumab plus T-VEC; one patient completed 2 cycles of ipilimumab plus nivolumab along with T-VEC and was able to restart single-agent nivolumab after the resolution of an immune-related adverse event (irAE); one patient received nivolumab and T-VEC. Pembrolizumab was injected every 3 weeks; nivolumab was given every 2 weeks; the ipilimumab and nivolumab combination was given every 3 weeks.

Throughout the follow-up period, 7 patients experienced irAEs. Most of the irAEs were mostly attributed to checkpoint inhibitor therapy. Two patients experienced grade 3 transaminitis; one patient had grade 3 hypophysitis; one patient developed grade 3 nephritis; one patient experienced grade 3 diarrhea. Grade 1 and 2 irAEs include 1 incidence of macular edema and 2 incidences of pruritic rash. No Grade 4 or 5 adverse events were observed in any patient. All patients reported occasional chills and fevers that were attributed to T-VEC injection. irAEs led to the suspension of the checkpoint inhibitor therapy in four patients prior to the complete resolution of their tumors.

### Outcome

**Fig. 3:**
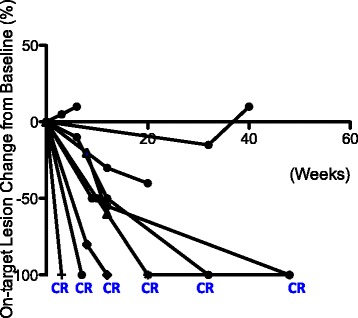
Change of tumor burden in on-target lesions. Change of tumor burden from baseline (before T-VEC injection) in injected lesions for each patient is shown as a function time measured in weeks. The time of T-VEC injection is set at 0. End of line indicates time of observation

**Fig. 4:**
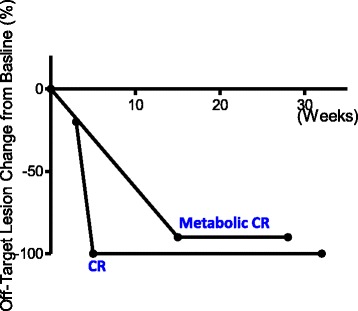
Change of tumor burden in off-target lesions. Changes of tumor burden for two patients who had measurable uninjected lesions are shown as a function of time measured in weeks. The time of T-VEC treatment is set at 0. End of line indicates time of observation

The ORR to treatment in this cohort was 90% (Table 1 and Fig. 2). The median time to response was 8 weeks (Figs. 1 and 3). 60% (6/10) of patients experienced CR and 30% (3/10) of the patients achieved partial response in the T-VEC injected, on-target lesions (Figs. 2 and 3). Two patients had off-target lesions in the lung, both of whom experienced CR in their uninjected pulmonary metastases (Fig. 4).

**Fig. 5:**
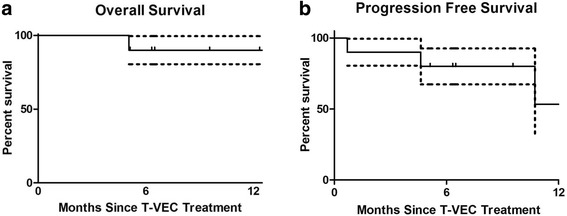
Overall and Progression Free survival. (**a**) 12-month overall survival. (**b**) 12-month progression free survival. Data expressed as time since T-VEC injection (*n* = 10). Dotted line represents ± standard of error

Three patients had progression of disease: one patient progressed through T-VEC injection; two patients achieved partial response before eventual disease progression (Figs. 1 and 3). In one case, disease progression appeared to be associated with the suspension of checkpoint inhibitor therapy (Figs. 1 and 3), suggesting that anti-PD-1 may serve an important maintenance function in sustaining response durability. Two patients died during follow-up. One patient died from disease progression and the other died from an unrelated cause (Fig. 5a and b).

### Changes in circulating T cells

**Fig. 6:**
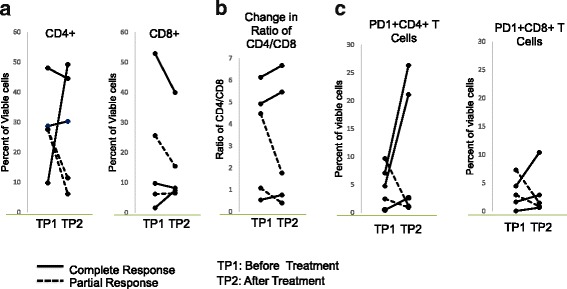
Changes in circulating T cells. (**a**) Percentages of circulating CD4 and CD8 T cells in complete and partial responders responders before and after T-VEC plus pembrolizumab (**b**) CD4:CD8 ratios complete and partial responders responders before and after T-VEC plus pembrolizumab. (**c**) Percentages of PD1+ CD4 and CD8 T cells in complete and partial responders responders before and after T-VEC plus pembrolizumab

As this study was not based on a clinical trial, we started collecting blood midway through the series and obtained pre-treatment specimens on 5 of the patients, including 3 complete responders and 2 partial responders. To assess the impact of the combined treatment of T-VEC plus an anti-PD1 based regiment on T cells, we analyzed blood samples from these 5 patients before and 3 months after treatment. Changes in the frequencies of peripheral blood CD4 and CD8 T cells were not associated with T-VEC plus anti-PD1 based therapy (Fig. 6a). However, the CD4:CD8 ratio was elevated in all 3 complete responders but not in any of the partial responders (Fig. 6b). In addition, the percentages of PD1^+^ CD4 T and CD8 T cells, a population reported to contain tumor-reactive lymphocytes [10], were greatly increased after the T-VEC plus anti-PD1 based therapy in all complete responders but not in partial responders (Fig. 6c).

## Discussion

Limited by a retrospective analysis with short follow-up time and small sample size, we could not reliably estimate the median survival and 12-month survival rate for this group of patients. However, 80% of the patients were still alive at the end of the follow-up. Importantly, we observed a particularly high CR of 60% (Fig. 2). Since a previous study has shown that CR is strongly associated with long-term survival in melanoma patients [11], it is possible that the patients in this cohort may have a favorable survival outcome.

The ORR of 90% and CR of 60% observed in our study are higher than data reported for stage IIIB-IV1a patients in the OPTiM trial (ORR 40.5, CR 16.6%) [4], data reported for the phase Ib T-VEC + Ipilimumab trial (ORR 50%, 22%) [2], and recently published results from the Phase 1b portion of the MASTERKEY-265 study (ORR 62%, CR 33%) [9]. The higher response rates in our cohort might be attributable to the patients who received nivolumab or nivolumab plus ipilimumab (Fig. 1) in our study. Future clinical trials are required to determine which checkpoint inhibitor therapy synergizes optimally with T-VEC injection. In addition, the phase 1b design of MASTERKEY-265 administered pembrolizumab 5 weeks after initiation of T-VEC as opposed to our cohort, in which the majority of patients were started on checkpoint inhibition either before or simultaneously with TVEC injection. It has been suggested sequencing of treatments might be important for optimal synergy, but little clinical data exist regarding what the ideal sequence would be. Interestingly, we had two patients who had prior exposure to checkpoint inhibition before T-VEC injection, and both patients achieved complete responses. Differences between response rates in our cohort and the phase Ib MASTERKEY-265 cohort may in some part be attributable to the earlier timing of checkpoint inhibitor therapy. Certainly the timing of checkpoint inhibitor therapy in combination with TVEC will require further investigation to derive the best outcome from the combined treatment.

Interestingly, among the 6 patients that achieved complete response, three patients saw a slower tumor regression. We reviewed the clinical parameters of both the three patients that showed more rapid complete response and those that saw a slower tumor regression. Parameters including stage of the disease, lesion location (cutaneous versus subcutaneous lesion), timing of treatment and prior treatment history were considered in the comparison. However, due to the limited sample size, we could not pinpoint a clear common feature for either group. Factors that affect the kinetics of response to T-VEC treatment have yet to be examined in details. T-VEC is a genetically modified version of HSV-1. Therefore, HSV-1 seropositivity may affect the response to the treatment. Unfortunately, HSV1 seropositivity was not tested for the patients in our cohort. Although previous studies did not find any association between HSV-1 serostatus and response durability or overall survival, it would be interesting to assess whether presence of antibodies against HSV-1 may delay the response to T-VEC in future studies.

It has been shown that TVEC contributes to anti-PD-1 immunotherapy by augmenting the inflammatory state of the tumor microenvironment (TME), which results in the increased homing and activation of tumor reactive T cells whose activity is prolonged by PD-1 blockade [12, 13]. Promoting the influx of T cells into the tumor is particularly important for patients that initially can’t respond to PD-1 blockade due to their low intratumoral TIL numbers [8]. Indeed, intratumoral administration of single agent T-VEC resulted in increased levels of circulating and tumor infiltrating T cells [3, 9]. When combined with anti-PD1 therapy (MASTERKEY-265 study) T-VEC responses were independent of baseline CD8+ infiltration, PD-L1 status or IFN-γ signature, and were instead associated with increased intratumoral inflammation, characterized by enhanced CD8+ T cell infiltration and elevated IFN-γ gene expression [9]. These results support the premise that TVEC can favorably alter the TME to facilitate T cell activity in response to PD-1 blockade.

In addition to promoting T cell infiltration, TVEC might also contribute to the priming of T cells by increasing the availability of both tumor antigens and antigen presenting cells [8]. It was recently reported that effective anti-PD1 therapy is dependent on an active co-stimulatory signal delivered through CD28 [14]. That study highlighted the importance of appropriate co-stimulation, which T-VEC might also facilitate by providing a GM-CSF/dendritic cell differentiation signal and by increasing the availability of antigens.

Consistent with these findings, following combinatorial treatment with TVEC and PD-1 blockade we observed elevated levels of PD-1-expressing, circulating T cells among complete responders as compared to partial-responders. Expression of PD-1 on peripheral T cells identifies tumor reactive T cells [10]; thus elevation of this sub-population could suggest enhanced intratumoral priming in response to TVEC and PD-1 blockade, as well as systemic anti-tumor effects as previously reported in the MASTERKEY-265 trial. Interestingly, in that trial combination TVEC/anti-PD-1 therapy did show modest increases in PD-1 positive CD8 T cells, in contrast to our study in which changes were more evident. This discrepancy might be attributable to the disproportionate number of complete responders evaluated. It also might be due to the small patient cohort we evaluated, and the variability of the treatments we employed. Further evaluation of our patients and those in other ongoing trials will clarify these findings.

In addition to T-VEC, several new oncolytic virus based therapies are under clinical development. Notably, a phase Ib study (NCT02307149) is investigating the efficacy and safety of Coxsackievirus A21 (CVA21) combined with ipilimumab in patients with unresectable Stage IIIB/C-IVM1c melanoma [15]. Preliminary results from the study reported similar ORR for the CVA21 plus ipilimumab [16] as that reported for the T-VEC plus ipilimumab [11]. Although both CVA21 and T-VEC both are virus-based oncolytic therapy, CVA21 is based on a human enterovirus associated with respiratory tract infections while T-VEC is engineered from a herpes virus strain. Our data are provocative for the hypothesis that the choice and timing of checkpoint inhibitor therapy may improve treatment efficacy. Additionally the type of oncolytic therapy may also be a key clinical decision when designing combination regimens in the future.

Toxicity is a major consideration for choosing optimal checkpoint inhibitor therapy to combine with oncolytic treatment. About 26% of the patients experienced grade 3 or grade 4 treatment-related adverse events in the phase 1b trial for the combined treatment of T-VEC and ipilimumab, much higher than the 11% reported for single agent T-VEC reported in the OPTiM trial [3]. In the recently published phase Ib results of the MASTERKEY-265 study, the combination arm of T-VEC and pembrolizumab did not increase toxicities as compared to the monotherapy arm [9]. While we did not observe any grade 4 or 5 immune-related adverse events in our cohort, 40% of our patients had grade 3 events during follow-up, including 2 patients who were briefly treated with combination ipilimumab and nivolumab (Fig. 1). Considering the overall higher toxicity associated with ipilimumab-associated regimens, combination therapies with anti-PD-1 agents as the backbone checkpoint inhibitor in conjunction with an oncolytic viral agent are sensible and expected for the foreseeable future.

Before the advent of oncolytic-virus based therapies, the only locoregional treatment for patients with unresectable melanoma was isolated limb perfusion (ILP), or isolated limb infusion (ILI) [17]. ILP or ILI therapy is limited to the limbs and provides no systemic benefits [18]. T-VEC plus checkpoint inhibition combines systemic treatment with locoregional therapy to offer both palliation and long-term disease control in all anatomic locations. Ongoing studies are reporting encouraging outcomes especially with regard to the ORR. In conclusion, the combined therapy of T-VEC and checkpoint inhibition represent a promising therapeutic option for patients with unresectable melanoma.

## Conclusion

In this report, we reviewed 10 cases of unresectable stage IIIB to stage IV melanoma patients who were treated with a combination therapy of T-VEC and checkpoint inhibitor for unresectable disease. 90% ofpatients saw response in their on-target lesions (Table 1). 60% of patients achieved CR in on-target lesions (Table 1). Two patients in this cohort had distant metastases to lung. Both patients saw CR in these off-target lesions (Fig. 4). Our data suggest that combining checkpoint inhibitor(s) with T-VEC injection may provide a synergistic efficacy for patients with unresectable melanoma. We observed a better overall response rate and complete response rate compared to published studies on similar therapeutic regimens.
